# Supplementary material for: Analysis of Viral and Host Factors on Immunogenicity of 2018, 2019, and 2020 Southern Hemisphere Seasonal Trivalent Inactivated Influenza Vaccine in Adults in Brazil
Source: Viruses. 2022 Jul 30;14(8):1692. doi: 10.3390/v14081692 (PMC9413331; doi:10.3390/v14081692)
Supplement: Supplementary file 1 [file viruses-14-01692-s001.zip › viruses-1784727-supplementary.pdf]

Supplementary Table S1: Impact of other populational variables on GMT-Post# and SP-Post# in 2019

			H1N1		H3N2		Influenza B	
			H1/CA	H1/MI	H3/SI	H3/SW	B/PH	B/CO
Comorbidity	Y	GMT-Post* (corrected) (Log2)	3.7 (1.3)	3.7 (1.1)	1.8 (1.2)	3.8 (1.4)	2.2 (1.2)	3.3 (1.5)
		SP-Post (GMT-Post*>corrected cutoff) (%)	96	96	84	94	96	87
	N	GMT-Post* (corrected) (Log2)	4.3 (1.7)	4.0 (1.6)	2.5 (1.8)	4.3 (1.8)	2.5 (1.5)	3.4 (1.7)
		SP-Post (GMT-Post*>corrected cutoff) (%)	93	91	86	95	83 *	84
Recent Flu	Y	GMT-Post* (corrected) (Log2)	3.8 (1.3)	3.9 (1.2)	2.0 (1.6)	4.7 (1.5)	2.4 (1.4)	3.3 (1.8)
		SP-Post (GMT-Post*>corrected cutoff) (%)	94	94	81	100	94	75
	N	GMT-Post* (corrected) (Log2)	4.0 (1.5)	3.8 (1.4)	2.1 (1.5)	3.9 (1.6)	2.3 (1.4)	3.4 (1.5)
		SP-Post (GMT-Post*>corrected cutoff) (%)	94	94	85	94	81	97
Sex	F	GMT-Post* (corrected) (Log2)	3.9 (1.5)	3.8 (1.3)	2.2 (1.5)	4.0 (1.5)	2.2 (1.3)	3.3 (1.6)
		SP-Post (GMT-Post*>corrected cutoff) (%)	86	95	88	97	81	85
	M	GMT-Post* (corrected) (Log2)	3.9 (1.6)	3.8 (1.4)	1.8 (1.5)	3.8 (1.8)	2.6 (1.3)	3.6 (1.5)
		SP-Post (GMT-Post*>corrected cutoff) (%)	92	92	79	87 *	87	89

Statistical significance is represented as * for $p<0.05$.
